# Electro-Acupuncture Stimulation Improves Spontaneous Locomotor Hyperactivity in MPTP Intoxicated Mice

**DOI:** 10.1371/journal.pone.0064403

**Published:** 2013-05-30

**Authors:** Haomin Wang, Xibin Liang, Xuan Wang, Dingzhen Luo, Jun Jia, Xiaomin Wang

**Affiliations:** 1 Neuroscience Research Institute, Peking University, Key Laboratory for Neuroscience of the Ministry of Education, Beijing, PR China; 2 Department of Neurology and Neurological Sciences, Stanford University, Stanford, California, United States of America; 3 Department of Physiology, Capital Medical University, Key Laboratory for Neurodegenerative Disorders of the Ministry of Education, Beijing, PR China; National Institute of Health, United States of America

## Abstract

Bradykinesia is one of the major clinical symptoms of Parkinson`s disease (PD) for which treatment is sought. In most mouse models of PD, decreased locomotor activity can be reflected in an open field behavioral test. Therefore the open field test provides a useful tool to study the clinic symptoms of PD patients. Our previous work demonstrated that 100 Hz electro-acupuncture (EA) stimulation at ZUSANLI and SANYINJIAO protected the dopaminergic nigrostriatal system of C57BL/6 mice from MPTP toxicity, indicating that acupuncture might be an effective therapy for PD sufferers. In the present study, we investigated the effects of 100 Hz EA stimulation on the spontaneous locomotor activity in MPTP injured mice. Here we found that, in MPTP treated mice, the total movements significantly decreased and the movement time, velocity and distance dramatically increased, although the dopaminergic nigrostriatal system was devastated, revealed by immunohistochemistry and HPLC-ECD. After 12 sessions of 100 Hz EA stimulation, the total movements elevated and the movement time, velocity and distance decreased, in MPTP mice. 100 Hz EA increased striatal dopamine content in MPTP mice by 35.9%, but decreased its striatal dopamine turnover. We assumed that the injury of other regions in the brain, such as the A11 group in diencephalon, might be involved in the hypermotility in MPTP mice. The effects of 100 Hz EA on spontaneous locomotor activity in MPTP mice might not relate with the striatal dopamine, but with its neuroprotective and regulatory effects on motor circuits in the brain. Our study suggests that EA might be a promising treatment for neurological disorders including PD.

## Introduction

Parkinson`s disease (PD) is the second most frequently diagnosed neurodegenerative disease in the elderly. Its cardinal symptoms are resting tremor, rigidity, bradykinesia and postural instability, and its main pathological changes are profound loss of dopamingergic neurons in the substantia nigra pars compact (SNpc) and dopamine in the striatum. Although tremendous efforts have been made in the treatment of this disease, a long-term, effective therapy is still lacking.

Acupuncture is a branch of Traditional Chinese Medicine, which has been extensively and safely practiced in curing diseases in China for over 3,000 years. In recent decades, some studies reported that acupuncture could alleviate motor disorders, reduce the dosage of anti-Parkinsonian drugs and relieve non-motor problems of PD patients [1]–[4], while some other studies reported acupuncture had no effects on motor disorders of PD patients [5]–[7]. The controversies may be due to the difference in the treating schemes, acupuncture protocols, variable symptom profiles of patients involved and small sample sizes, etc. [5], [6], [8].

Compared with the clinical debates, animal experiments were in agreement with the claims that acupuncture was effective for PD by showing that acupuncture protected the dopaminergic nigrostriatal system or improved the abnormal movements in different kinds of PD animal models, i.e., the MFB-transected rat model, the 6-OHDA lesioned rat model, the MPTP lesioned mouse and rhesus monkey model [9]–[14].

Among all these models, the monkey MPTP model is considered the best since MPTP is known to cause Parkinsonism in humans. However, the mice MPTP model is more popularly used to value the anti-Parkinsonian therapies. Our previous studies showed that electro-acupuncture (EA) at 100 Hz could protect the dopaminergic nigrostriatal system from MPTP injury in mice via anti-oxidative effects [11]. Here, we investigated the impacts of 100 Hz EA on spontaneous locomotor activities in the same model and found that 100 Hz EA could normalize the abnormal behavior of MPTP mice.

## Materials and Methods

### Ethics Statement

All experimental procedures were approved by the Committee on Animal Care and Usage of Capital Medical University in which all the principles followed the Chinese Specifications for the Production, Care and Use of the Laboratory Animals. All animal experiments were performed by Haomin Wang whose permit number of the License for Performing Animal Experiments of Beijing is 12928. All efforts were made to minimize animal suffering.

### Animals

Male C57BL/6J mice, weighting 22∼25 g, 8 weeks old, were supplied by the Laboratory Animal Center of Peking University, and housed in a temperature-controlled room (23±1°C) under 12-h on/off light cycle with food and water *ad libitum* in the home cage. They were allowed to acclimate to the breeding environment for 7 days before experiments.

### Subacute MPTP Mice Model

**Figure 1:**
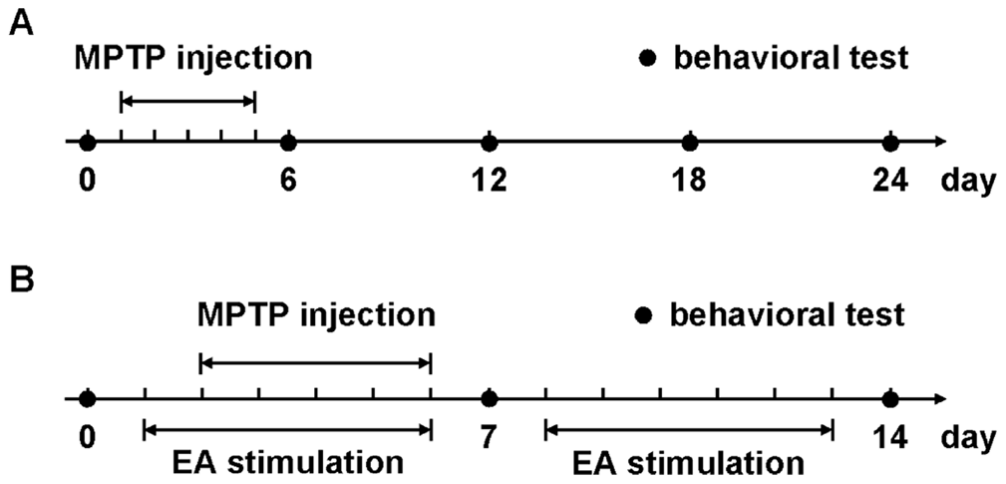
Experimental design of the study. (A) Subacute MPTP mice model. (B) EA stimulation on MPTP mice. Numbers represent days.

In the first cohort, fifty-five mice were randomly divided into a saline (NS) group (eleven mice) and a MPTP group (forty-four mice). As shown in Figure 1A, from day 1 to day 5 mice received intraperitoneal injections of MPTP (Sigma-Aldrich, St. Louis, MO, USA, 30 mg/kg, dissolved in saline) or an equivalent volume of saline once a day. Behavioral tests were performed on day 0, 6, 12, 18 and 24, and all mice in the NS group and eleven mice in the MPTP group were involved. On day 6, 12 and 18, eleven mice not participating behavioral test from MPTP group were sacrificed. On day 24, after behavioral test, the remaining mice were sacrificed.

### EA Stimulation on MPTP Mice

In the second cohort, fifty-one mice were divided into four groups randomly: NS (twelve mice), saline plus EA stimulation at 100 Hz (100 Hz+NS, twelve mice), MPTP plus EA stimulation at 0 Hz (0 Hz+MPTP, thirteen mice) and MPTP plus EA stimulation at 100 Hz (100 Hz+MPTP, fourteen mice). As shown in Figure 1B, the EA stimulation was performed from day 1 to day 13 except day 7, and from day 2 to day 6, mice were received MPTP or saline (the same strategy with the first cohort of mice) half-hour after EA treatment. On day 0, 7 and 14 all mice underwent behavioral tests. EA stimulation was performed as described before [11].

### Open Field

Locomotion activity was assessed as previously described [15], using Mouse Tru Scan system (Coulbourn Instruments, USA). Briefly, each mouse was in a dark closed individual cage (25.4 x 25.4-inch-square) with a grid of infrared beams mounted horizontally every 2.5 cm, and spontaneous locomotor activity was recorded as total movements, total movement time and total movement distance across the 60 min recording period. All the assays were started at 9∶00 am, and during the tests, the environment was kept quiet.

### Tissue Collection and Processing

For immunohistochemistry analysis, four mice from each group on one time point were sacrificed, then the brains were removed and tissues were prepared according to the previously methods [11].

For monoamine level evaluation, seven mice from each group on one time point in the first cohort and nine to eleven mice from each group on day 14 in the second cohort were decapitated, and the bilateral striata were dissected quickly and stored at −80°C.

### Immunohistochemistry and Quantification of TH-ir Neuronal Profiles

All sections spanning the SN were collected for immunohistochemistry according to the previously described method [11].

Systemic MPTP administration caused symmetrically bilateral lesions in the SNpc. Here, TH-ir neuronal profiles with distinct nuclear shapes in the left SNpc were counted in ten sections throughout the entire rostrocaudal extent of the left SNpc. All sections were coded and examined blind.

### HPLC Analysis of DA and its Metabolites

Striata collected were used to detect the levels of DA and its metabolites, dihydroxyphenylacetic acid (DOPAC) and homovanillic acid (HVA), by HPLC with electrochemical detection (HPLC-ECD) according to the previously described method [11].

### Statistical Analysis

Striatal monoamine levels were analyzed by one-way ANOVA followed by Least-Significant Difference (LSD) post hoc test of difference between means.

For the mouse behavior study, each animal’s movement was measured at different time points, i.e. day 0, 6, 18 and 24. The day 0 mean value of each group was set as baseline. A ratio was obtained by normalizing the mean value of each time point to its own value at day 0 (the baseline). The ratios between each group were compared statistically. Statistical analyses of spontaneous locomotor activity were carried out using repeated measures ANOVA with groups as the independent variables and time as the repeated measure. When between-subjects (group) effects were significant, comparisons between the groups on each time point were performed using univariate ANOVA (in the first cohort) or univariate ANOVA with LSD post hoc test (in the second cohort).

All statistical analyses were performed by SPSS 13.0. Values are expressed as mean ± SEM. In all cases, the null hypothesis was rejected at the 0.05 level.

## Results

### MPTP Damages the Dopaminergic Nigrostriatal System

**Figure 2:**
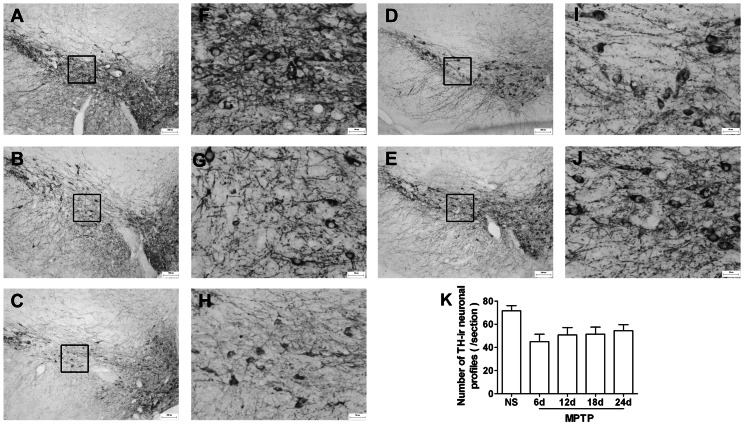
MPTP reduces the number of TH-ir neurons in the SNpc. (A and F) NS group. (B and G) MPTP group on day 6. (C and H) MPTP group on day 12. (D and I) MPTP group on day 18. (E and J) MPTP group on day 24. (K) Quantification of TH-ir neuronal profiles in the SNpc. Scale bar, 200 µm (A, B, C, D and E) and 50 µm (F, G, H, I and J). n = 3∼4.

To this day, MPTP is mainly used to destroy the dopaminergic nigrostriatal system of animals. As shown in Figure 2, on day 6 the number of TH-ir neurons in the SNpc of the MPTP-treated mice decreased to 62.8% of the normal amount, while from day 12 to day 24 a slight recovery occurred, keeping it at 70.8∼76% of the normal level.

**Figure 3:**
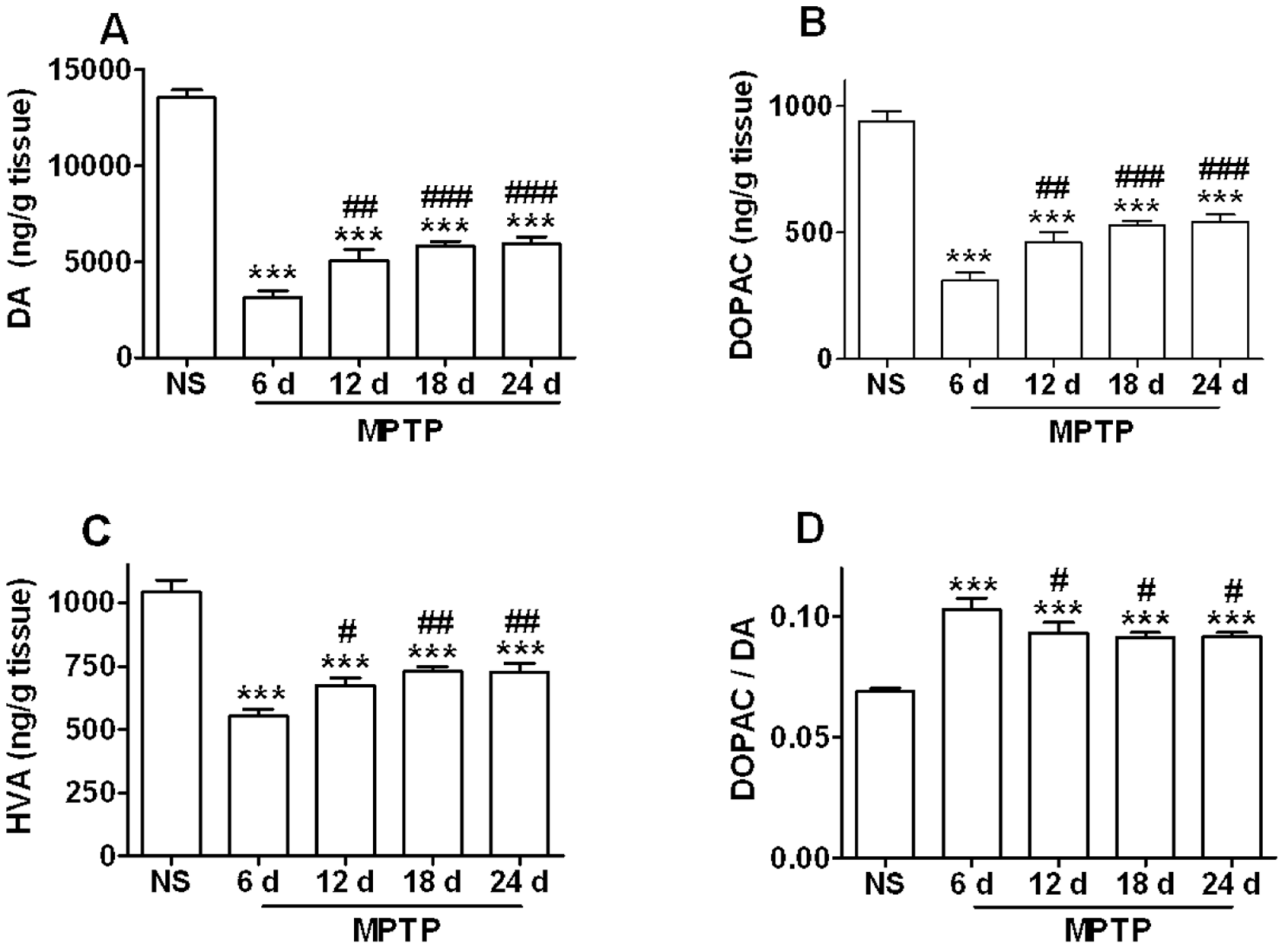
MPTP decreases striatal content of DA and its metabolites and increases the DA turnover. (A) DA. (B) DOPAC. (C) HVA. (D) DA turnover. *** *p<*0.001 *vs.* NS group; ^#^ *p<*0.05, ^##^ *p<*0.01, ^###^ *p<*0.001 *vs.* MPTP group on day 6. n = 6∼7.

MPTP dramatically decreased striatal DA and its metabolites levels (DA, df = 4, F = 94.987, *p* = 0.000, Figure 3A; DOPAC, df = 4, F = 49.789, *p* = 0.000, Figure 3B; HVA, df = 4, F = 26.128, *p* = 0.000, Figure 3C), and significantly increased the DA turnover (indicated by DOPAC/DA ratio, df = 4, F = 15.214, *p* = 0.000, Figure 3D), suggesting that DA insufficiency activated the compensatory pathways. Consistent with the tendency of TH-ir neurons in the SNpc, from day 12 on, the four indices recovered significantly compared with that on day 6 (DA and DOPAC, *p*<0.01, Figure 3, A and B; HVA and DA turnover, *p*<0.05, Figure 3, C and D). Taking DA for example, on day 6 it dropped to 23%, yet during day 12 to day 24 it was back to 37.4∼43.8%.

These data showed that in this model the dopaminergic nigrostriatal system suffered the severest injury when MPTP administration just completed (day 6), then recovered to some extent, and was in a relatively stable state from day 12 to day 24.

### MPTP Increases the Spontaneous Locomotor Activity

**Figure 4:**
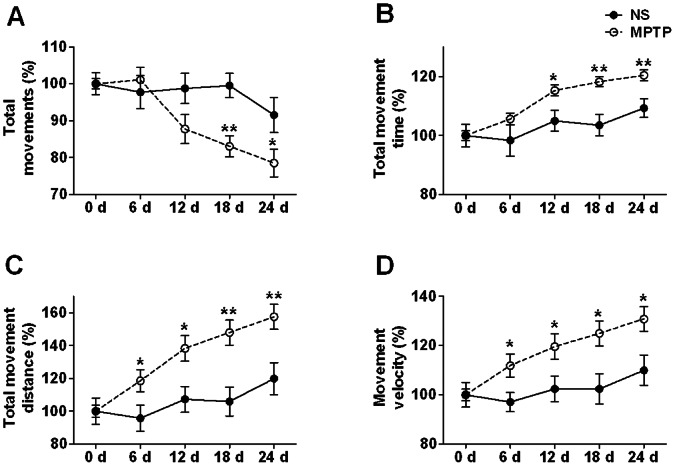
MPTP increases the spontaneous locomotor activity. (A) Total movements. (B) Total movement time. (C) Total movement distance. (D) Movement velocity. Filled circle with solid line represents the NS group, and hollow circle with dash line represents the MPTP group. *p<0.05, **p<0.01 *vs*. NS group on the same day. n = 10∼11.

One movement is defined as a series of successive coordinate changes without rest for at least one sample interval in the floor plane. MPTP significantly reduced the total movements in mice (df = 1, F = 5.662, *p* = 0.028, Figure 4A), suggesting either an increased movement time (hyperkinesia) or an increased rest time (hypokinesia). The total movement time in MPTP-lesioned mice markedly increased over time (df = 1, F = 8.975, *p* = 0.007, Figure 4B), indicating that the reduction of total movements in MPTP intoxicated mice was due to hyperkinesia not hypokinesia. Elevated total movement time contributed to the dramatically augmented total movement distance in MPTP mice (df = 1, F = 16.164, *p* = 0.001, Figure 4C). Movement velocity was acquired by total movement distance normalized with total movement time, and also notably raised by MPTP in mice (df = 1, F = 14.357, *p* = 0.001, Figure 4D). On day 24, the total movement time was, on average, 10% higher, movement velocity was 19% higher and total movement distance was 31% higher for MPTP mice compared with the control mice, exhibiting a phenomenon of hyperkinesia.

### 100 Hz EA Improves the Abnormal Behavior of MPTP Mice

In our previous study, 0 Hz EA stimulation did not show any influence on Parkinsonian mice and rats [11], [16]. In this study, we used 0 Hz+MPTP group as a control because puncturing the acupoints on the legs might affect the motion of animals.

**Figure 5:**
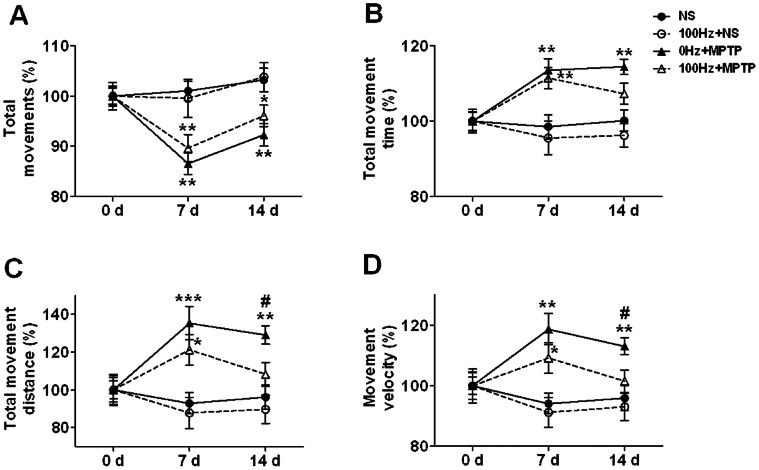
100 Hz EA stimulation improves the hyperkinesia of MPTP mice. (A) Total movements. (B) Total movement time. (C) Total movement distance. (D) Movement velocity. Filled circle with solid line represents the NS group, hollow circle with dash line represents the 100 Hz+NS group, filled triangle with solid line represents the 0 Hz+MPTP group, and hollow triangle with dash line represents the 100 Hz+MPTP group. *p<0.05, **p<0.01, ***p<0.001 *vs*. NS group on the same day; ^#^ *p*<0.05 *vs*. 100 Hz+MPTP group on the same day. n = 12∼14.

On day 14, total movements in 0 Hz+MPTP group and 100 Hz+MPTP group were still significantly lower than that in NS group (*p* = 0.002 and *p* = 0.038 respectively, Figure 5A), while total movement time in MPTP mice received 100 Hz EA administration recovered to some extent but had no statistical difference with that in the MPTP mice (*p* = 0.061, Figure 5B).

100 Hz EA had more prominent effects on movement velocity of MPTP mice, which was significantly reversed on day 14 (*p* = 0.033 *vs*. 0 Hz+MPTP group, Figure 5D). Both decreased total movement time and movement velocity accounted for the dramatically reduced total movement distance on day 14 in MPTP mice received 100 Hz EA administration (*p* = 0.02 *vs*. 0 Hz+MPTP, Figure 5C). In addition, 100 Hz EA stimulation did not influence the spontaneous locomotor activity of normal mice (Figure 5).

### Effects of 100 Hz EA on Striatal DA and its Metabolites

**Figure 6:**
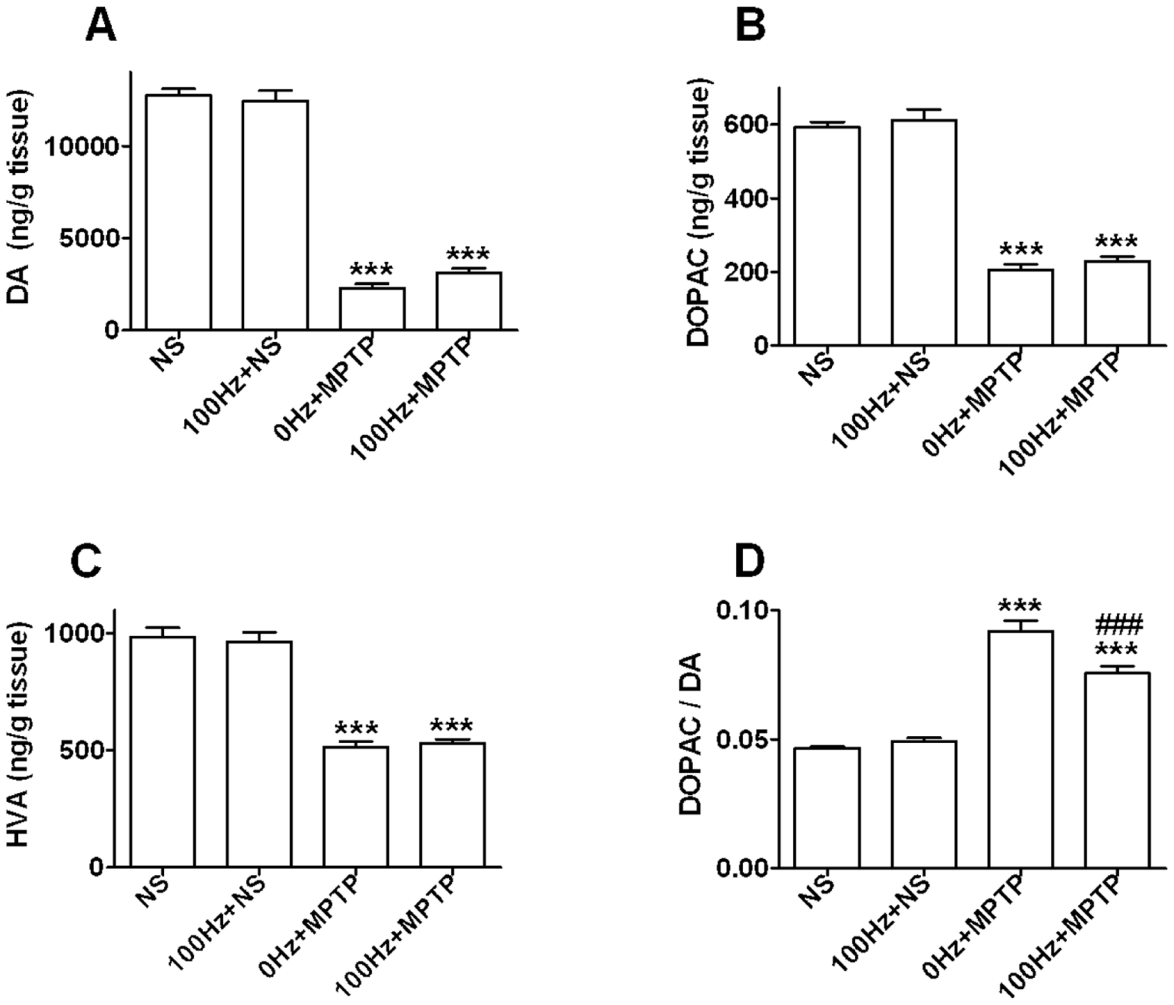
Impacts of 100 Hz EA stimulation on striatal DA and its metabolites. (A) DA. (B) DOPAC. (C) HVA. (D) DA turnover. ***p<0.001 *vs.* NS group; ^###^ *p<*0.001 *vs.* 0 Hz+MPTP group. n = 9∼11.

On day 14, MPTP dramatically decreased the contents of DA, DOPAC and HVA but increased DA turnover in the striatum (*p* = 0.000 *vs.* NS group, Figure 6). Although the DA level was 35.9% higher in the 100 Hz+MPTP group than that in the 0 Hz+MPTP group, the difference was not significant (*p* = 0.106, Figure 6A). However, DOPAC and HVA levels in MPTP mice were not enhanced in such amplitude by 100 Hz EA treatment (Figure 6, B and C). Therefore, its DA turnover was significantly decreased (*p* = 0.000 *vs.* 0 Hz+MPTP group, Figure 6D). In addition, 100 Hz EA stimulation did not have effects on striatal DA and its metabolites in normal mice (Figure 6).

## Discussion

In this study, we observed that in a commonly used mouse PD model, although the dopaminergic nigrostriatal system was severely damaged by MPTP, the spontaneous movement time, velocity and distance increased. After stimulating acupoint ZUSANLI and SANYINJIAO with 100 Hz EA, we found that the locomotor hyperactivity in this model was normalized. 100 Hz EA increased striatal DA content in MPTP-lesioned mice, but not DOPAC and HVA content, making the DA turnover decreased. It suggested that striatal DA might not play a role in the effect of 100 Hz EA on the spontaneous locomotor activity. In addition, 100 Hz EA did not affect the striatal DA and its metabolites as well as the spontaneous locomotor activity in normal mice.

MPTP-lesioned C57BL/6 mice model is widely used to assess novel anti-Parkinsonian therapies, but conflicted results show decreased, increased and neutral phenomenon on locomotor activity, which might be due to different sources of mice or intoxication protocols [17], [18].

In our study, striatal DA in MPTP mice remained 23% at its lowest level (day 6), and was about 40% when hyperkinesia became dominant (from day 12 to day 24). It is commonly assumed that a 70∼90% DA deficiency is required for symptom appearance in PD [19], and a striatal DA loss greater than 80% is necessary to observe symptoms across species [17]. In our previous study on an acute MPTP C57BL/6J mice model (15 mg/kg four times at 2 h interval, i.p.), striatal DA was left 7.7%, and the spontaneous locomotor activity markedly decreased [15]. Therefore, the “suprathreshold” injury of the dopaminergic nigrostriatal sytem might contribute to the hyperkinesia.

Aside from the open field test we had also used the rotarod test, a test widely used to measure coordinated motor skills [17]. We also observed an upward tendency in MPTP mice compared with the normal control. However, no statistical difference existed between groups (n = 10 each group, data not shown).

MPTP is highly lipophilic and crosses the blood-brain barrier soon after the systemic injection. In the brain, it is transformed into the toxic form, MPP^+^, in non-dopaminergic cells. MPP^+^ has a high affinity for the dopamine transporters (DAT) through which it enters the DA neuron to kill the host cell. Apart from the SNpc, there distribute DA cells in other regions of the central nervous system, such as the DA A11 group in the caudal diencephalon, which projects into the spinal cord, forming the diencephalospinal pathway. Perturbation of the A11 group is thought to be involved in restless legs syndrome (RLS) [20], a common disease characterized by an intense urge to move the limbs, which can be mitigated by dopamine replacement therapy. There also exists the A11 diencephalospinal pathway in C57BL mice [21], and 6-OHDA can lead to locomotor hyperactivity in mice by damaging the A11 group [22]. MPTP is also able to destroy the A11 group in non-human primates [23]. In our model, the A11 group might be injured and contribute to the hyperkinesia. Thus, only if it is very severely damaged (e.g. >80% loss of striatal DA), can the motor effects resulting from the devastated dopaminergic nigrostriatal system become dominant and hypokinesia emerge. In addition, our data does not suggest that this model is a potential RLS model, because contrary to it, in the SN of RLS patients there is an increased TH and no cell loss [24].

Furthermore, MPTP can destroy other monoaminergic or non-monoaminergic regions that do not express DAT [23]. Rousselet and colleagues assumed that hyperkinesia in MPTP mice might be due to the disturbance of prefrontal cortex [18]. In Rommelfanger`s study, the hypokinesia did not appear in MPTP treated mice until norepinephrine loss occurred [25], however, this could not help to explain the hypermotility in MPTP mice in our study.

The mechanism of 100 Hz EA in normalizing hyperkinesia in MPTP mice is still unclear. Multiple-mechanisms might be involved in the whole process. Our previous studies showed that 100 Hz EA eased oxidative stress in this model [11], while others demonstrated that acupuncture stimulated neurotrophic factor release, and had anti-inflammatory and anti-apoptotic effects [10], [12], [26]–[28], which might also contribute to prevent neuronal damage from MPTP in this study. Our previous studies on PD rats showed that 100 Hz EA could balance neurotransimtters or neuropeptides in the motor circuits. In Jiàs study, 100 Hz EA increased GABA content and substance P content in the midbrain to improve the abnormal behavior of PD rats [16], [29]. In Huòs study, it was suggested that the cortex might play the most important role in the regulation of internal balance in PD rats after 100 Hz EA treatment [9]. In addition, Huang et al. reported that acupuncture increased regional cerebral blood flow in the frontal lobe and the basal ganglia of PD patients [30].

Collectively, hyperkinesia in MPTP mice may reflect the multiple injury patterns of MPTP. Acupuncture might exert extensive neuroprotective and regulatory effects on motor circuits in PD brain.
